# Heat Stability Assessment of Milk: A Review of Traditional and Innovative Methods

**DOI:** 10.3390/foods13142236

**Published:** 2024-07-16

**Authors:** Jianfeng Wu, Simin Chen, Paul Van der Meeren

**Affiliations:** 1 College of Food Science, South China Agricultural University, Guangzhou 510642, China; jianfeng.wu@scau.edu.cn; 2 Faculty of Bioscience Engineering, Ghent University, 9000 Ghent, Belgium; paul.vandermeeren@ugent.be; 3 School of Pharmaceutical Sciences, Guangzhou Medical University, Guangzhou 511436, China

**Keywords:** heat stability, milk, assessment method, traditional technique, innovative technique

## Abstract

It is important to differentiate milk with different thermostabilities for diverse applications in food products and for the appropriate selection of processing and maintenance of manufacturing facilities. In this review, an overview of the chemical changes in milk subjected to high-temperature heating is given. An emphasis is given to the studies of traditional and state-of-the-art strategies for assessing the milk thermostability, as well as their influencing factors. Traditional subjective and objective techniques have been used extensively in many studies for evaluating thermostability, whereas recent research has been focused on novel approaches with greater objectivity and accuracy, including innovative physical, spectroscopic, and predictive tools.

## 1. Introduction

Fresh bovine milk at a natural pH is considered heat stable; it can withstand heating at 140 °C for more than 10 min without coagulation or gelation. However, compositional differences between milk samples might affect its heat stability. Moreover, with the diversification of the application of milk and its components, milk is subjected to a variety of processing and storage conditions, as well as different chemical environments, such as acidic, alkaline, or Ca^2+^-fortified conditions [1,2,3]. For the discrimination of milk samples with different thermostabilitities for different applications, it is of great importance to have reliable methods to assess the heat stability. Furthermore, heat-induced coagulation of milk poses a high risk to the production equipment and quality of the consumer product, as well as the shelf-life consideration of the product. Therefore, effective assessment or prediction of the thermostability of dairy products will be important for the selection of processing and maintenance of manufacturing facilities. The heat stability of milk is hereby defined as the ability of milk to withstand high temperatures without visible coagulation or sediment formation during storage [4]. Various assessment methods have been developed, including traditional methods (subjective and traditional objective methods) and innovative methods using innovative physical, spectroscopic, or predictive tools [5,6,7]. The determination of the heat coagulation time (HCT) of milk is a subjective method. This widely used method is regarded as a standard method in many countries and regions of the world owing to its low cost and easy operation. However, some inconsistency might be introduced due to its limitations, such as the subjectivity in visual detection of heat coagulation and the discrepancy in experimental performance (e.g., different heating ramp conditions). Some conventional subjective methods, such as determination of the amount of soluble (insoluble) protein upon centrifugation, are more scientific and accurate, but most of these are time-consuming and labor-intensive. Recently, a variety of innovative methods with a combination of high objectivity and less requirement of time and labor were proposed by researchers.

Dumpler, Huppertz, and Kulozik [8] previously reviewed the traditional techniques for the assessment of the heat stability of milk and their effects on the heat stability. However, a comprehensive review of traditional and innovative techniques has never been addressed. In this work, a brief description of the basic mechanism of heat-induced coagulation of milk is provided first, and then the emphasis is given to the traditional, and more importantly, state-of-the-art, strategies for determining milk heat stability and their influential factors. Ultimately, the potential perspectives are overviewed.

## 2. The Mechanisms of Heat-Induced Coagulation of Milk

The heat stability assessment is normally performed at a temperature of 140 °C for milk with normal concentration and at 120 °C for concentrated milk until gel-like coagulation is observed. A better understanding of the mechanisms of coagulation when milk is subjected to high temperatures helps in designing a more scientific assessment of the heat stability of milk.

In the process of heat stability evaluation, heat-induced coagulation of normal milk is widely considered to be a result of the instability of colloidal casein micelles rather than the polymerization of proteins. Protein polymerization is, most likely, a concomitant intra-micellar reaction during heating [9]. It was proposed by H. Singh [10] that in the initial stages of the heat coagulation, a change in colloidal interactions was involved to allow casein micelles to approach each other for chemical reactions to occur.

It is widely agreed that the colloidal stability of casein micelles depends mostly on the C-terminus brush of the κ-casein [9,11]. The heat-induced dissociation of κ-casein or the collapse of κ-casein due to heat-induced acidification both make a significant contribution to the instability of casein micelles. Without the protection of the κ-casein brush, the micellar casein is prone to aggregate in the presence of sufficient calcium ion activity [12,13]. During the heat coagulation process, whey proteins (WPs), especially β-lactoglobulin (β-lg), denature and further aggregate with each other or associate with κ-casein by disulphide bonds. It is commonly agreed that the interaction between whey protein and κ-casein is pH-dependent in milk with a normal concentration; whey protein prevents the dissociation of κ-casein from micelles at a pH < 6.7, whereas it facilitates the κ-casein dissociation at a pH > 6.9 [14]. It has not been elucidated how whey protein monomers or aggregates pass through the hairy layer to find the disulphide bonds of κ-casein yet. In one of the explanations, κ-casein in the hairy layer is thought to change during heating, or a rearrangement of the micelle surface may occur to facilitate this interaction [10]. Dephosphorylation of caseins is another important change during heating [6,15]. The phosphate groups in casein amino acid residues can interact with colloidal calcium phosphate (CCP) and provide negative charges for electrostatic repulsion to maintain the stability of micelles. Hence, dephosphorylation of casein damages the stability of the casein micelles.

There are a large number of changes in the serum phase contributing to heat coagulation. A significant decrease in pH happens during heating, which exerts a destabilizing effect on milk [15]. This heat-induced decrease in pH is primarily owing to the acid produced from lactose oxidation, the hydrolysis of organic phosphate groups, and the precipitation of calcium phosphate [16]. Additionally, calcium phosphate is transferred from the serum phase to the colloidal phase, resulting in the instability of colloidal casein micelles due to the shielding of their negative charges [17].

Concentrated milk is commonly known to be less heat-stable as compared to unconcentrated milk. A kinetic model of heat-induced coagulation of concentrated skim milk with a total solid content of 12–33% was established by Dumpler, Peraus, Depping, Stefánsdóttir, Grunow, and Kulozik [18] using a Weibullian model. Hereby, heat coagulation is considered as a two-step process, including a destabilization phase of casein micelles and a following aggregation phase, which both depend on the milk environment and the heating conditions. The polymerization of proteins is not the dominant factor in the coagulation of unconcentrated milk, whereas the concentration of whey protein in concentrated milk is high enough to form a complex network in milk, resulting in a visible coagulum, and hence has to be taken into account [19]. It was observed that the coagulum primarily consisted of aggregates of denatured WPs as well as those associated with casein micelles (at pH < 6.5). At a higher pH, the coagulum is probably formed by WPs and dissociated casein in the serum phase [15]. Moreover, the concentration has a remarkable effect on the κ-casein dissociation [10]. In concentrated milk, it was found that the dissociation of micellar κ-casein happened within an extensive pH range. As compared to unconcentrated milk, the extent of κ-casein release in concentrated milk is higher at the same pH, and the critical pH shifts toward a lower pH. Consequently, κ-casein dissociation occurs at the native pH of concentrated milk (pH 6.5–6.6). Dumpler and Kulozik [20] obtained a deeper insight into heat-induced coagulation of concentrated skim milk via direct steam injection (DSI) heating, which eliminated the heat-induced acidification in this fast heating and cooling process. It was proved that the dissociation of κ-casein from micelles was more prone in concentrated milk, and a loss in structural integrity of the casein micelles was possibly a step before the formation of a coagulum.

The heat-induced changes in milk are expected to be different through different heating modes, including indirect heating, direct steam injection, and direct steam infusion. Hereby, indirect heating means heat transfers occur by contacting the milk with a metal surface that is heated by steam or superheated water on the other side, whereas, in a direct heating system, superheated steam is injected into a stream of milk (i.e., steam injection) or that milk is sprayed into (i.e., steam infusion). It was reported that, in comparison with indirect heating, self-aggregation of β-lactoglobulin preferably occurs at higher temperatures in direct steam injection, which leaves less β-lactoglobulin available to associate with casein micelles [21]. Regarding the average particle size of casein micelles, UHT by steam injection, but not indirect heating and direct steam infusion, led to a significant increase in casein micelle diameter, indicating an effect of the hydrodynamic forces in steam injection [22]. However, so far, no studies have indicated clear differences between direct and indirect heating on the mechanism of heat coagulation of milk.

**Figure 1:**
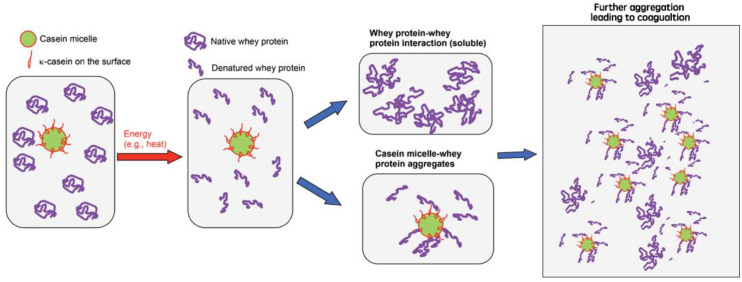
Schematic diagram of heat coagulation of milk (adapted from with permission from Ref. [23]).

A Schematic diagram of heat coagulation of milk can be found in Figure 1 (adapted from Patel et al. [23]).

## 3. Factors Influencing the Heat Coagulation of Milk

As the most widely adopted indicator of the assessment of milk heat stability, heat coagulation is affected by a large number of factors, including the assessment conditions, the milk composition, and the milk processing conditions. During the process of the assessment, several factors should be considered to minimize the experimental error. Additionally, a number of compositional and processing factors in a milk sample can play an important role in its heat stability. Therefore, the conditions in the heat stability test for different samples (like temperature and sample volume) should be adjusted accordingly, and some factors could be used as indicators to predict heat stability.

### 3.1. Influencing Factors in Assessment

Davies et al. [5] investigated the influencing factors in the subjective evaluation setup, including sample volume, head space gas, rotation speed, and heating temperature. It was found that the HCT decreased as the proportion of head space oxygen to milk volume increased until a constant minimum time was recorded at a critical value of the proportion. Similar results were observed in a study by Darling [24]. The experiments should thus be carried out with enough headspace to exclude these effects. Changing the heating temperature (from 105 °C to 140 °C) greatly affected the HCT (375 min vs. 2.9 min). In the relationship between the logarithm of the HCT and the temperature, milks could be classified into two groups: those that were less heat-stable were characterized by a curvilinear relationship (the slope slightly decreased with increasing temperature), and those that were more heat-stable were characterized by a fairly constant change in the plot of log HCT against temperature. A study by Prakash, Kravchuk, and Deeth [25] indicated that a similar coagulum amount was found at heating temperatures of 135 °C and 145 °C, but heating at 150 °C induced much more coagulum. In an evaluation of the heat stability of recombined evaporated milk by Kasinos, Karbakhsh, and Van der Meeren [26], it was found that a period of 9–10 min was needed for recombined concentrated milk (10 mL) to reach the required heating temperature of 121 °C. With stirring by a metallic propeller stirrer in the oil bath to keep the temperature uniform, positioning in the oil bath and the cooling medium were found to have no significant effect on the measurement.

Darling [24] demonstrated that the use of reconstituted milk from low-heat-treated skim milk powder (SMP) rather than natural milk could have effects on the HCT. Several environmental variations, such as light, could also influence the results’ reproducibility. Thus, the experiments should be carried out under controlled artificial lighting to exclude this effect.

The heating medium might have an effect on the assessment of the heat stability of milk. During indirect heating via an oil bath, some chemical changes occur in milk, like heat-induced acidification due to lactose degradation, phosphate release from phosphoserine residues in casein, and Maillard reaction products, and hence facilitate the instability of milk in addition to the heat-induced direct effects. However, these chemical changes were found to not be correlated with the onset of coagulation. In a study by Dumpler et al. [20], direct steam injection (DSI) was applied to the heat coagulation measurement, which eliminated the interfering chemical changes and hence offered the opportunity to obtain a more scientific heat stability assessment and investigate the mechanisms of the heat-induced coagulation process owing to the fast heating and cooling process via DSI. Lee, Barbano, and Drake [27] also demonstrated that heating by DSI caused less damage in skim milk than indirect heating with lower values in the amount of serum protein denaturation, furosine, and color.

### 3.2. Compositional and Processing Factors

Compositional factors, including the initial pH, ionic composition, milk standardization materials, and indigenous plasmin activity are reviewed below. The initial pH of the samples prior to heat stability analysis plays a significant role in heat stability testing [28]. Hence, it is significant for heat stability studies that the initial pH values of the samples are reproducible and the variation should be kept in a range of ±0.02 pH units. Therefore, it was suggested that titration curves should be obtained by measuring the pH against the titrant amount, and more accurate pH values were obtained from the smooth fit curve [24]. Ho et al. [29] proposed that the type of pH adjusting agent could play a role in the measurement of the heat stability as well. It was found that adjusting the pH of a liquid milk protein concentrate using citric acid resulted in a lower viscosity in milk during heating, because of avoiding the release of Ca^2+^ from the colloidal phase to the serum phase by using HCl. A more detailed discussion of pH effects on milk heat stability can be found in the previous studies [10].

Generally, the natural variation of salt in milk is too small to be considered [30]. However, the manual adjustment in salt composition can play a role in the heat stability of milk. Calcium supplementation of milk increases the Ca^2+^ concentration, but also decreases the milk pH considerably, which both lead to a lower heat stability. Ramasubramanian, D’arcy, and Deeth [31] demonstrated that there was a direct Ca^2+^ effect on the heat coagulation of milk proteins, except from the Ca^2+^-induced pH decreasing effect, when over 50 mM Ca^2+^ was added. Addition of ionic calcium at concentrations above 20 mM resulted in the coagulation of milk at 70 °C for 5 min, even at the normal pH of milk (6.6), and up to 98% of the protein was coagulated in preheated milk with the addition of 50 mM CaCl_2_ at this heating condition. Faka, Lewis, Grandison, and Deeth [32] proved the strong effect of variations in Ca^2+^ concentration in reconstituted SMP on its heat stability via a reduction of the Ca^2+^ concentration; samples containing a slightly higher Ca^2+^ concentration than the threshold exhibited a poor heat stability. Hence, Ca^2+^ concentration can even be used as an indicator of the heat stability of milk and reconstituted SMP. The different ionic compositions of the reconstituted MPC originating from MPC35 (35% protein) to MPC90 (90% protein) with a constant 8.5% protein content were found to have a strong effect on the HCT [33]. Less dissociation of κ-casein after heating was found in samples originating from MPC with a higher protein content; thus, the heat stability increased from MPC35 to MPC70, although MPC80, MPC85, and MPC90 had a very poor heat stability due to the high concentration of ionic calcium.

The standardization material of edible lactose powder (ELP) versus permeate powder (PP) from skim milk ultrafiltration was shown to have obvious effects on the heat stability of reconstituted SMP. At a pH of 6.3–7.0, a higher heat stability was found for reconstituted low-heat-standardized SMP containing PP as compared to reconstituted milk containing ELP [34].

Indigenous plasmin activity could markedly affect the heat stability–pH profile of dairy products, probably by reduced zeta-potential of the casein micelles [35]. It was reported that plasmin activity markedly affected the heat stability–pH profile of raw skim milk and serum protein-free milk. By contrast, the effect of plasmin on the heat stability of pre-heated milk was less pronounced, shifting the heat stability–pH profile to the more alkaline side, while the heat stability of concentrated milk was nearly unaffected by plasmin [36].

Except for compositional factors, the heat stability of milk is also greatly affected by different types of processing, such as preheating and homogenization. It is commonly considered that the pre-heating of milk prior to evaporation/concentration increases the heat stability of milk. SMP can be classified according to the severity of pre-heating and the content of native whey protein [37]. Normally, more severe pre-heating favors the heat stability of reconstituted milk [38]. During pre-heating, WPs denature, followed by their interaction with κ-casein, which can provide steric hindrance for casein micelles against heat-induced aggregation [14,39]. Liang, Patel, Matia-Merino, Ye, and Golding [40] studied the impact of pre-heat treatment (90 °C for 5 min) on the heat stability of MPC-stabilized emulsions. It was found that emulsions stabilized by preheated MPC had a slightly better heat stability, and the presence of non-micellar casein had positive impacts on preventing WPs from aggregation. Nonetheless, severe pre-heating could cause a negative effect on the heat stability as the preheating achieves sufficient whey protein denaturation but insufficient WP–casein interactions in some cases, which leads to easier heat-induced gelation [41]. Homogenization can also alter milk thermostability. According to studies by McCrae and Muir [42], the heat stability of recombined milk decreases with increasing severity of homogenization (i.e., homogenization pressure from 5.2 to 20.7 MPa) at pH 6.7, but weaker effects of homogenization were found at pH conditions far from 6.7. As the homogenization pressure increased, the fat globule size was reduced, and hence more proteins were adsorbed at the fat globule surface. A lower HCT was obtained with a higher proportion of protein adsorbed onto the interface between the fat and aqueous phases. Meena, Singh, Borad, and Panjagari [43] also found a detrimental effect of homogenization on the heat stability of skim milk ultrafiltered retentate.

## 4. Quantitative Assessment Methods for Heat Stability of Milk

### 4.1. Traditional Assessment Methods for Heat Stability of Milk

The traditional quantitative assessment of the heat stability of milk can be classified into subjective and objective methods according to the manner of recognition of the indicator, which is mostly heat-induced coagulation.

#### 4.1.1. Subjective Assessment Methods

The traditional subjective methods include the test of the heat coagulation temperature, as well as the classical test of heat coagulation time (HCT). The former is a method for determining the temperature at which the coagulation appears. This method is mostly adopted in some specific cases, such as low pH. An approximately linear relationship between pH in the range of 5.4–6.2 and coagulation temperature was observed [44]. However, its application is limited due to the time-consuming measurement at various temperatures.

The HCT test, which is most widely used nowadays, has been developed by Davies et al. [5]; it determines the time elapsing between putting a small glass vial containing the milk sample in a thermostatically controlled oil bath and the onset of visible coagulation of the milk. In their set-up, a milk sample (2.5 mL) is placed into a 4.0 mL glass tube (8.75 mm diameter × 122 mm length), and the tube is sealed with a silicone–rubber stopper and clamped on a carriage, which is then held horizontally and immersed into hot liquid paraffin (135 °C). The tube can rotate with the carriage at a variable speed (8 cycles/min). As soon as a clot is observed by microscopy in the samples, the heat coagulation time (HCT) is recorded. The accuracy of HCT determination via this method was found to be 2.5% [24].

**Figure 2:**
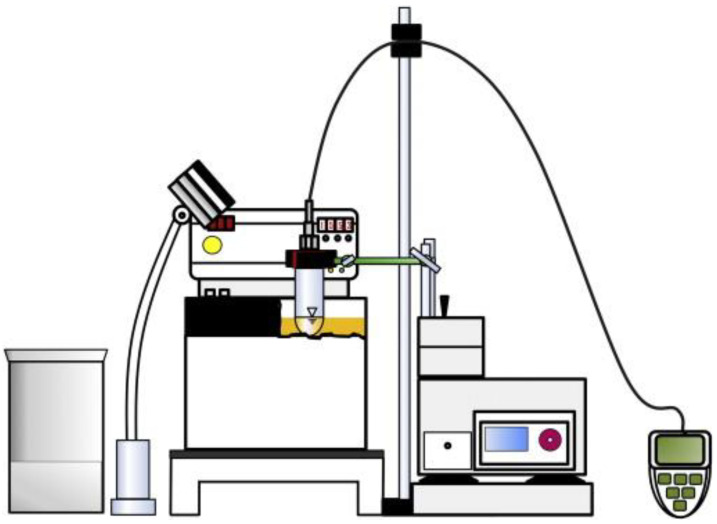
Set-up of a subjective heat stability test developed by Dumpler and Kulozik. It consists of an oil bath, an ice-water bath, a lamp, a shaker equipped with a support for fixation of the sample-containing tubes, a lamp, and an ALMEMO^®^ 2590-4S data logger (Ahlborn Mess- und Regelungstechnik, Holzkirchen, Germany) (reproduced with permission from [45]).

Dumpler and Kulozik [45] used a modified set-up in terms of a more instant and better visibility of coagulation based on the abovementioned method for the determination of the heat stability of concentrated skim milk with a solid content up to 35% (Figure 2). In this set-up, the level of the sample was equal to the oil bath level, and the tubes were shaken at a speed of 300 min^−1^, instead of rotating with the carriage. In this way, the centrifugal forces induced by shaking made a thin film of sample on the glass surface above the filling level of the tubes. It was thus easy to detect coagulation, which reduced the subjectivity of detecting the onset of coagulation. A clear correlation between the heat stability of unconcentrated skim milk and its corresponding concentrates was shown via this method, indicating that the heat coagulation time and temperature of skim milk with limited compositional variation could therefore be used to predict the heat stability of the corresponding concentrated milk with a certain total solid content (up to 35%).

#### 4.1.2. Traditional Objective Methods

Due to the inherent limitation of subjectivity in the classical test by visible observation, efforts have been made to develop objective methods using some traditional tools. These subjective methods can generally be divided into 2 categories, including monitoring via the remaining soluble (insoluble) protein and the particle size/viscosity of milk.

As early as 1966, White and Davies [46] proposed an objective method for the determination of heat sensitivity. They used a similar setup to that in the subjective method but removed the samples at predetermined intervals and immediately cooled them down in water. The samples were subsequently subjected to centrifugation for 30 min at 300× *g*. Finally, the total nitrogen in the supernatant was determined by the Kjeldahl method and a plot of the total nitrogen in the supernatant against heating time was obtained. In most samples, there was an induction period in these plots during which a very limited amount of aggregated protein was detected, followed by a sharp increase that preceded visible coagulation by only 1–2 min. Finally, about 80% of the protein was found to be aggregated, 15% was converted to proteose-peptone and non-protein nitrogen, and 5% remained suspended in the aqueous phase. This method had a good correlation with the subjective method. Dumpler et al. [20] adapted their subjective test by using direct steam injection (DSI) with a minimum of subjectivity and less variable results. They set up the relationship between the total solid content of concentrated milk, temperature, and heating time on the onset of heat coagulation in a pilot scale. In this research, the coagulation was determined by scanning electron microscopy (SEM), particle size analysis, and the amount of sedimentable protein after centrifugation; 2% of the sedimentable protein measured after centrifugation could be seen as the onset of heat coagulation by comparison with SEM and particle size analysis. Chen, Grandison, and Lewis [47] described a heat stability test of goat milk via determining the mass of sediment: heated milk was well shaken and then centrifuged at 2760× *g* for 30 min. After removal of the supernatant, the sediment was dried at 105 °C until a constant weight for determination of the dry weight.

**Figure 3:**
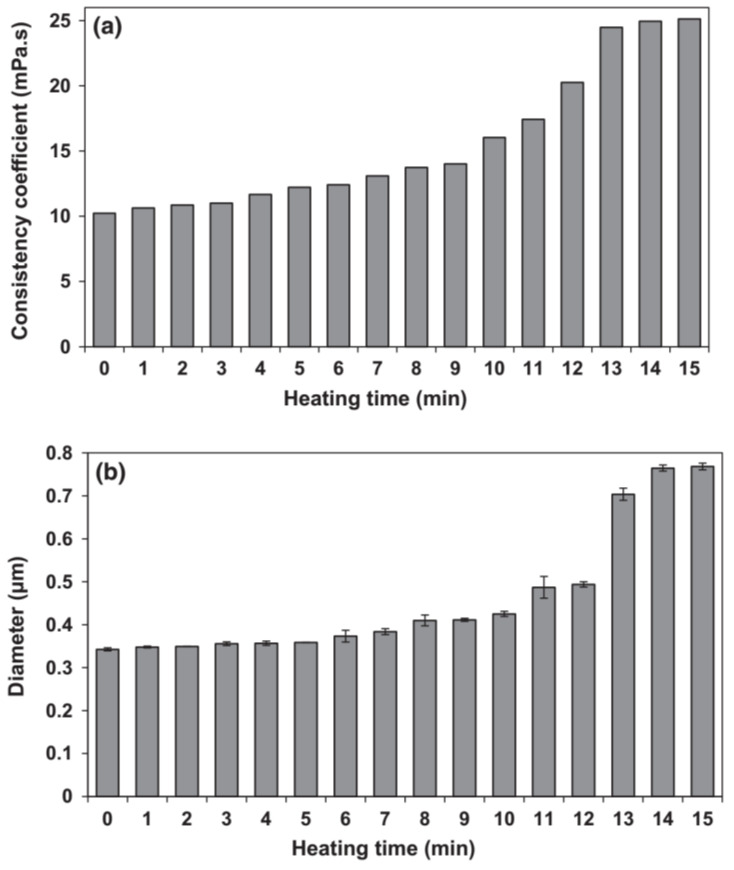
Changes in consistency coefficient (**a**) and volume-weighted average diameter (**b**) of recombined concentrated milk as a function of time during heating at 121 °C (reproduced with permission from [26]).

The stability indices of milk, such as particle size and viscosity, can be used to evaluate the heat stability of milk [48]. Kasinos et al. [26] developed a small-volume objective evaluation method for the heat stability of REM via both viscosity and particle size analyses. Both parameters increased more than two-fold when coagulation occurred (Figure 3). Ho et al. [29] found a correlation between viscosity, particle size data, and HCT. Meza, Zorrilla, and Olivares [49] developed a heat-induced aggregation evaluation method for calcium-enriched milk at low temperature (25–80 °C) by a rheometric test. Samples were tested with a rotary speed-controlled rheometer with a cone-plate geometry. A clear increased tendency in viscosity was observed when aggregation, induced by colloidal instability, occurred. Liang, Matia-Merino, Patel, Ye, Gillies, and Golding [50] compared the heat coagulation of milk-protein-concentrate (MPC) emulsions via three objective methods (particle size, microstructure, and rheological measurements) and the subjective HCT measurement. Close to HCT, the particle size distribution of the emulsions changed from bimodal to multi-modal, indicating the aggregation of both casein micelles and oil droplets. The viscosity index, determined as the viscosity after a heat treatment relative to the viscosity before heat treatment, was used as an indicator of coagulation. The three objective measurements showed good correlations with the heat stability determined by HCT. It was concluded that coagulation occurred, accompanied by a sharp increase in viscosity index and a marked jump in particle size of aggregates as well as an open gel-like structure with increased void area. It was observed from the SEM results in the study by Dumpler et al. [20] that the coagulum was composed of large spherical proteinaceous particles (10–20 μm), and even larger non-spherical particles composed of spherical particle aggregates. At the surface of these spherical particles, casein micelles merged into the particle surface could be observed. An increase in the average particle size of non-aggregated micelles was observed as well as a broadening of the particle size distribution before the start of visible coagulation; the appearance of particles ranging from 3 to 100 μm represented the protein aggregates.

The heat stability of milk can be determined by these objective indices in a more accurate way. However, the procedure of these measurements is labor-intensive and time-consuming, which limits their practical applications.

### 4.2. Innovative Assessment Methods for Heat Stability of Milk

Due to the limitation of conventional methods, innovative methods with both higher objectivity and less labor and time for assessing heat stability have recently been proposed.

#### 4.2.1. Innovative Physical Methods

**Figure 4:**
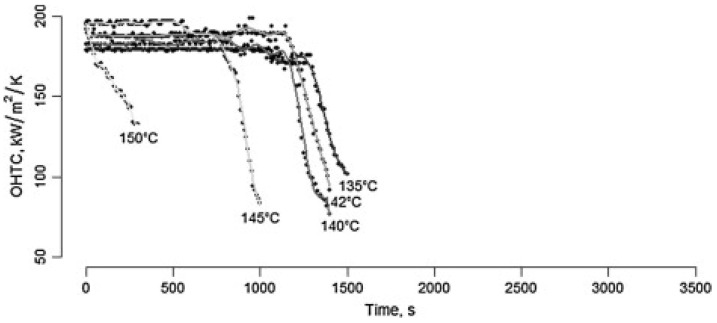
Changes in *OHTC* of reconstituted skim milk with pre-heating to 65 °C for 5 s when subjected to heat treatment at various temperatures (reproduced with permission from [25]).

Prakash, Datta, and Deeth [51] reviewed physical methods for the determination of milk fouling caused by heating via the changes in acoustic properties, capacitance, electrical resistance, and thermal resistance, which was potentially useful for the detection of heat coagulation. Among these, it was reported that thermal resistance could be used to monitor the heat-induced fouling of milk in real time, which was determined by subtracting the convection resistance (the resistance at the beginning of the run) from the total resistance (i.e., fouling resistance plus convection resistance). A higher thermal resistance was found due to the proteinaceous deposit with low density [52]. This method was applied to determine thermal denaturation and subsequent fouling of whey proteins [53,54] and milk [55] in a plate heat exchanger. Zhang, Xu, Villalobos-Santeli, and Huang [56] compared the heat-induced fouling of camel milk and bovine milk by monitoring the heat transfer coefficient. Another method for the determination of UHT stability of milk protein products was proposed by measuring the changes in overall heat transfer coefficient (*OHTC*) [57]. This technique has been applied to monitor the heat stability of chocolate-flavored milk [58], milk protein concentrates with mineral salt addition [59], and high-protein milk dispersions [60]. The plot of *OHTC* against heating time is used to monitor the development of coagulation/fouling during UHT heat treatment. If coagulation occurs, the *OHTC* shows a sharply decreasing tendency (Figure 4). The *OHTC* can be calculated as shown below:

(1) $$OHTC=\frac{GC_{p}\Delta \theta }{A\Delta T_{lm}}$$

*G* is the mass flow rate of the dispersion in kg/s; $C_{p}$ is the specific heat of the dispersion in J/kg °C; Δ*θ* is the temperature difference between the inlet and outlet of the UHT section, in °C; *A* is the heat-exchanging surface area of the tubing in m^2^, and Δ*T_lm_* is the log mean temperature difference.

Hong-liang et al. [61] quantitatively evaluated the thermal stability of skim milk using multiple light scattering (Turbiscan^®^, Warsaw, Poland). With this technique, no sample pre-treatment is needed, and the back-scattered light and transmitted light intensity can be timely obtained after heat treatment. Hence, heat-stability indications, like the migration velocity of the particles, and the thickness of the sediment layer can be calculated. Among these, the instability index (*ISI*) is an important parameter, which can be calculated as specified below:

(2) $$ISI=\sqrt{\frac{{\sum_{i=1}^{n}{(x}_{i}-x_{BS})}^{2}}{n-1}}$$

where $x_{i}$ is the backscattered light intensity of the sample obtained by a single measurement at a certain height (%), $x_{BS}$ is the average value of $x_{i}$ measured after the sample is scanned from top to bottom, and *n* is the total number of scans.

After the heat treatment, the backscattered light intensity at the bottom typically shows an obvious increase, while the backscattered light intensity at the top decreases, indicating an obvious particle precipitation phenomenon in milk, which leads to an increase in the particle concentration at the bottom and a decrease in the particle concentration at the top. The heat stability of milk can be obtained according to the instability index at a certain time after heating. Based on multiple light scattering, the Turbiscan^®^ can be used to quickly evaluate the heat stability of milk samples and to screen the optimal heat treatment conditions during manufacturing.

Protein denaturation normally occurs before heat coagulation of milk, which thus can possibly serve as indicator of heat stability via detection using differential scanning calorimetry (DSC) and thermogravimetric analysis (TGA). A thermal transition between 60 °C and 80 °C is typically reported in milk protein products (e.g., milk protein isolate (WPI) and milk protein concentrate (WPC)) using DSC, indicating unfolding of whey proteins, and a second thermal transition can be observed between 105 °C and 115 °C, which corresponds to the thermally induced interaction of denatured whey proteins with the caseins [62]. TGA is a technique for displaying the weight loss profile of the sample upon heating. Khalesi and FitzGerald [63] reported that for WPC, the first peak was between 40 °C and 120 °C, and the second started at 250 °C and ended at 400 °C. The former region was related to protein denaturation and aggregation and moisture lost from the samples, while the second region was related to sample decomposition.

#### 4.2.2. Spectroscopic Methods

A large number of chemical reactions in milk are involved during the heat coagulation of milk, including structural and conformational changes in the proteins (e.g., protein denaturation and interactions), the Maillard reaction, and alteration of heat-labile vitamins. The measurement of the fluorescence spectra of some milk compounds (e.g., tryptophan, riboflavin, vitamin A, and advanced Maillard reaction products) using fluorescence spectroscopy has been proven to enable the monitoring of the heating process of milk before or at the onset of coagulation.

Most studies determining milk coagulation using fluorescence spectroscopy focused on rennet- or acid-induced coagulation. However, changes in the fluorescence spectra of many milk compounds are due to changes in the environment, like protein conformational changes or interactions, which happen during heat-induced coagulation as well. Therefore, these studies can be used as a reference for the development of heat stability assessment. Blecker, Habib-Jiwan, and Karoui [64] demonstrated that the measurement of fluorescence intensity at 466 nm, which was ascribed to the riboflavin component, using synchronous fluorescence spectroscopy (SFS), was highly sensitive to changes in the local environment; there was an inflection point in the fluorescence intensity which could be identified as the onset of coagulation. A good correlation was found between the gelation time determined by fluorescence and rheology (R^2^ = 0.9). The results clearly indicated that SFS has potential as a rapid and non-destructive technique for determining the changes in network structure and molecular interactions during the milk coagulation process. Fluorescence of tryptophan has also been found to allow the monitoring of the modification of proteins and of their physiochemical environment upon coagulation [65].

Before the coagulation occurs, some indicators were also able to assess the heat-treated milk. It was found that an increase in both intrinsic and 1-anilino-8-naphthalenesulfonate (ANS) fluorescence intensity of a β-lg solution by greater than 20% was caused by heat treatment at 80 °C for 15 min; the quenching constants of acrylamide and KI increased by 15.8% and 268% after heating at 75 °C for 15 min, indicating the increased accessibility of both Trp19 and Trp61 due to the conformational transition of β-lg during heating [66]. Croguennec, Mollé, Mehra, and Bouhallab [67] reported the ability of fluorescence spectroscopy combined with circular dichroism (CD) to characterize heat-induced, non-native β-lg monomers. It was found that heating induced β-lg dimers to transform into monomers with exposed, nonnative Cys119 in the molten globule-like state. Kamal and Karoui [68] studied the effects of heat treatment on camel milk using front-face fluorescence spectroscopy. The discrepancy in fluorescence spectra of vitamin A of milk with different heat-holding times and temperatures was observed due to its sensitivity to changes in the solvent viscosity, induced by protein–lipid interactions during heating. Boubellouta and Dufour [69] also studied the potential of synchronous front-face fluorescence spectroscopy for the characterization of milk changes during mild heating. The intensity of the band ascribed to vitamin A was decreased by 80% when the temperature of milk was increased from 4 °C to 50 °C. Parallel factor (PARAFAC) analysis revealed that the first three components explained 94.43% of the total variance for heating, with the first and second components corresponding to tryptophan and vitamin A, and the third component corresponding to riboflavin. These studies showed the potential of fluorescence spectroscopy to monitor and evaluate the heat stability of milk.

Diffusing wave spectroscopy (DWS) allows the determination of changes when aggregation of the micelles occurs by monitoring the apparent radius or the optical properties of the milk (e.g., turbidity parameter). DWS exhibited the ability to assess the coagulation of casein micelles, with a steep increase in both apparent radius and turbidity parameter [70,71]. The apparent radius rose sharply by more than a thousand-fold, while a 3-fold increase in the normalized turbidity was observed when aggregation of concentrated casein micelles occurred [71]. Alexander and Dalgleish [72] used DWS to study the interactions between denatured milk serum proteins and casein micelles in aggregating systems.

Near-infrared (NIR) spectra were found to be a useful tool for detecting the coagulation of milk. Wang et al. [73] developed a novel approach to identifying heat-induced physical and chemical modifications in milk by infrared spectroscopy coupled with the random forest model, with an accuracy as high as 0.97. Klandar, Lagaude, and Chevalier-Lucia [74] reported that the absorbance at 860 nm showed a sharp change of more than 100% during the onset of flocculation or gelation of reconstituted milk with calcium enrichment at a level of 6.25 mmol·kg^−1^; comparable coagulation parameters were found via dynamic small-amplitude oscillatory rheometry (DSAOR) and NIR spectra analysis. A good agreement was also observed between the coagulation time measured by a traditional mechanical device (Formagraph) and a NIR device in a study by Cipolat-Gotet, Cecchinato, De Marchi, Penasa, and Bittante [75].

Raman spectroscopy can be used to identify the conformational changes in proteins during thermal treatment, e.g., transformations of the tertiary and the secondary structures via the analysis of the amide I band of the protein [76,77]. Micro-Raman spectroscopy was used in the determination of heat-induced fouling deposits from a whey protein solution in a plate heat exchanger. Raman signatures of β-lg were recorded in the range of 800–1800 cm^−1^ toward the unfolded state and in aggregates upon heating [78]. Specific Raman signatures of protein aggregates were detected, which were distinct from the Raman spectra of unfolded β-lg solutions.

Acoustic and electroacoustic spectroscopy were reported to be superior compared to dynamic light scattering (DLS) for monitoring the effect of heating skim milk without requiring dilution or disruption [79]. Skim milk heated at 85 °C for 20 min was found to have a slightly higher values of attenuation compared to unheated milk for the skim milk samples concentrated more than twice, which might be attributed to the presence of heat-induced whey protein aggregates. The charge versus pH profile of casein micelles was pronouncedly affected by heating; the interaction of casein micelles with WPs during heating was concluded to be the reason for the increased surface charge density of the micelles.

**Figure 5:**
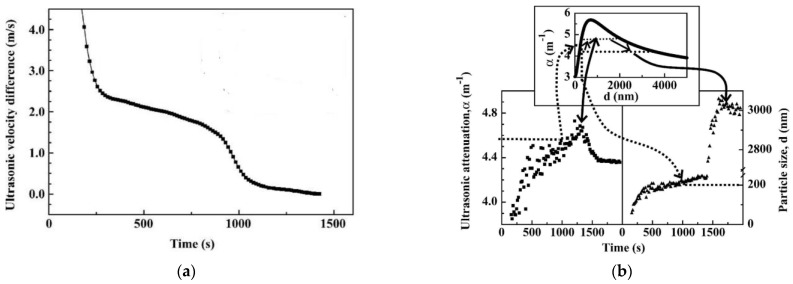
Profile of ultrasonic velocity difference in a non-preheated recombined evaporated milk sample upon heating at 120 °C at its natural pH (6.47) at 5 MHz (**a**); ultrasonic attenuation profile at 2.5 MHz of preheated recombined evaporated milk at pH 6.7 upon heating at 120 °C (bottom left), as well as the corresponding particle size profile (bottom right) and the plot of ultrasonic attenuation versus particle size (top) (**b**) (reproduced with permission from [7]).

The abovementioned techniques can provide quick and objective results. However, they are mainly useful for either the non-real-time monitoring of heat-induced coagulation or measurements for specified dairy products (e.g., calcium-enriched milk) at a lower temperature (<100 °C). To realize the real-time monitoring of heat-induced coagulation during heat stability assessment, several hindrances have to be overcome, including the requirement of high temperatures and prevention of boiling at a temperature above 100 °C during the measurement. High-resolution ultrasonic spectroscopy (HRUS) can be used in the analysis of opaque and highly concentrated materials as well as heterogeneous food systems [80]. It allows a broadly applied temperature range from −40 °C to 130 °C at elevated pressure and has the desired resolution for velocity and attenuation to reflect the molecular organization and molecular interactions [81]. Smyth et al. [81] introduced HRUS for determining the heat coagulation temperature of calcium fortified milk. The same coagulation temperature was found for both ultrasonic velocity and attenuation, with a resolution better than 0.1 K. Lehmann et al. [7] determined the heat coagulation of concentrated milk by HRUS. In the measurement, the milk sample was contained in a fitted, small-volume ultrasonic cell immersed in a temperature controller filled with tetraethylene glycol (120 °C). It was found that the pre-coagulation and coagulation processes could be separated into 3 stages according to the ultrasonic velocity and attenuation profiles. In the first stage, the denaturation and aggregation of WPs and precipitation of calcium phosphate caused a sharp decrease in ultrasonic velocity and increase in ultrasonic attenuation. Small changes in ultrasonic velocity were observed in the second stage. The third stage showed a sharp decrease in both ultrasonic velocity and attenuation, which was identified as the start of coagulation. Typical profiles of ultrasonic velocity and ultrasonic attenuation are shown in Figure 5. A good correlation was found between HRUS and the Irish Dairy Board subjective method [82]. Compared to particle size measurement, HRUS was proven to be an efficient real-time determination method for milk heat coagulation. HRUS also has the potential to realize real-time monitoring of heat-induced β-lg aggregation [83]. During the protein transition to the molten globular state and subsequent β-lg aggregate formation, there was a decrease in the ultrasonic velocity and an increase in compressibility. The former process showed no significant changes in ultrasonic attenuation, whereas a significant increase was observed in the latter. The evaluation of the effects of pH and heating rate on the aggregation size was proven applicable by ultrasonic spectroscopy as well.

#### 4.2.3. Prediction of Heat Stability of Milk

The prediction of the heat stability of untreated milk by associating its composition with technological characteristics can be a useful strategy to obtain milk thermostability in a rapid, low-cost, and non-destructive way and to monitor and discriminate milk quality more accurately before processing.

Some fast tests, such as the acid, alcohol, and phosphate tests, were used to predict the heat stability of milk in early studies, but it was found that they were not satisfactorily convincing to predict heat stability [24]. By using statistical analysis, Hanus et al. [84] evaluated the correlation between milk composition (i.e., fat (F); crude protein (CP); lactose monohydrate (L); solids non-fat (SNF); urea (U); residues of inhibitory substances (RIS); somatic cell count (SCC); total count of mesophilic microorganisms (TCM); count of coli-form bacteria (COLI)) and other indicators (i.e., milk freezing point (MFP)) with milk thermostability (TES) on a large data set (*n* = 2829). Weak positive correlations were found between the thermostability (TES) and log TCM or log COLI, with correlation coefficients of 0.169 and 0.124, respectively.

Mid-infrared spectroscopy (MIFS) can provide information regarding the interaction between physical matter and electromagnetic radiation in the region between 900 cm^−1^ and 5000 cm^−1^. MIFS can routinely provide information regarding the content of milk protein, fat and lactose, but also shows the ability to predict technological properties of milk, including its heat stability [85,86]. In the research of Visentin et al. [86] using MIFS combined with a model based on partial least squares (PLS) regression, it was found that the model fit statistics on the natural logarithm-transformed, predicted HCT were more accurate than the fit statistics using the back-transformed HCT. This model was considered as having a substantial predicting ability for the HCT, and was characterized by a concordance correlation coefficient (CCC) of 0.63. According to the predictions, the HCT had a relatively strong correlation with milk urea (correlation coefficient = 0.48) as well as a weak association with milk coagulation properties, with correlations from −0.22 to 0.34. Although this model could not be considered useful for analytical purposes (residual prediction deviation (RPD) < 2), this model could be used for screening purposes according to the CCC of prediction equations in external validation. Furthermore, if the number of records in the calibration data set used to develop the prediction model and the variability in the reference values is increased (sample number in this study = 713), the prediction accuracy of the models may improve. Frizzarin et al. [87] used modern statistical machine learning methods for prediction of milk quality traits from mid-infrared spectroscopy to improve the prediction accuracy. Their results indicated that the application of neural networks (i.e., nonlinear generalizations of a linear model) for regression analyses reduced the root mean square error by 3.67% compared with PLSR for heat stability. McDermott et al. [88] studied the prediction of the milk protein and free amino acid (FAA) composition using MIFS and their correlation with various technological properties, including heat stability. The prediction accuracy for external validation of the protein fraction was moderate, with the strongest correlation coefficient of total casein being 0.74, whereas a weak to moderate prediction accuracy was found for FAA (correlation coefficient from 0.51 to 0.75). The MIFS-predicted protein fractions/FAA were weakly to moderately correlated with the HCT of milk; Pearson correlations between protein fractions/FAA and the HCT ranged from −0.24 to 0.22 and −0.35 to 0.11, respectively. Additionally, Rocchetti and O’Callaghan [89] suggested the potential of applying metabolomics to assess milk heat stability based on GC-MS and LC-MS as well as NMR.

The heat instability of milk can be exhibited as a form of coagulation or gelation after heat treatment, but also as a form of invisible aggregates that precipitate during storage. Therefore, it is also important to predict the instability of milk during storage after heat treatment to avoid undesired sediment. Grewal et al. [6] established the correlation between spectral changes and instability development during storage of ultra-high-temperature-treated milk to predict sediment formation using attenuated total reflectance Fourier transform infrared spectroscopy (ATR FT-IR). Marker variables in the FT-IR spectra were identified as structural changes and interactions of proteins, carbohydrates, and fats. The correlation between the changes in spectral variables, e.g., in the ranges of 1800–1700 cm^−1^ (fat A) and 1700–1500 cm^−1^ (amide I and II), and sediment formation was established, which enabled the successful prediction of sedimentation in skim milk (R^2^ = 0.92) and whole milk (R^2^ = 0.60) by using the PLS regression technique.

## 5. Conclusions and Perspectives

This review focused onto different assessment methods that have been developed to evaluate the heat stability of milk. In general, traditional and innovative methods exhibit their specific advantages and limitations. It was found from recent studies that the traditional subjective method is still considered the most common and widely used technique to assess the heat stability of milk. Some objective methods can provide more scientific and accurate analysis, but they have obvious disadvantages, such as their time-consuming and labor-intensive operation. Several novel methods with both higher objectivity and less labor and time have been developed, but their relatively high investment cost and the requirement of professional operators highly limit their application. The prediction of heat stability using spectroscopy (e.g., mid-infrared spectroscopy) and modern statistics seems to be a promising strategy via the association of milk heat stability and its composition, but the predicting accuracy still needs to be further testified and improved. Hence, a larger sample database should be set up, and more systematic factor analysis (e.g., composition) should be performed in the future. Additionally, the heat stability of milk is affected by many factors. Hence, milk with different composition and treatment could possess an entirely different thermostability. Therefore, tailored assessment methods should be established for various dairy products with specific operational conditions. In summary, the traditional method, in particular, subjective observation by naked eyes, is still prevalent nowadays in most studies, but there is clearly a trend towards more objective and accurate methods.

## Data Availability

No new data were created or analyzed in this study. Data sharing is not applicable to this article.
